# Electroacupuncture Promoted Nerve Repair After Peripheral Nerve Injury by Regulating miR-1b and Its Target Brain-Derived Neurotrophic Factor

**DOI:** 10.3389/fnins.2020.525144

**Published:** 2020-09-29

**Authors:** Yu-Pu Liu, Zhi-rong Luo, Chang Wang, Hao Cai, Tian-tian Zhao, Han Li, Shui-jin Shao, Hai-dong Guo

**Affiliations:** Department of Anatomy, School of Basic Medicine, Shanghai University of Traditional Chinese Medicine, Shanghai, China

**Keywords:** electroacupuncture, brain derived neurotrophic factor, Schwann cells, peripheral nerve injury, miR-1b

## Abstract

Growing evidence indicates that electroacupuncture (EA) has a definite effect on the treatment of peripheral nerve injury (PNI), but its mechanism is not completely clear. MicroRNAs (miRNAs) are involved in the regulation of a variety of biological processes, and EA may enhance PNI repair by regulating miRNAs. In this study, the rat sciatic nerve injury model was treated with EA for 4 weeks. Acupoints Huantiao (GB30) and Zusanli (ST36) were stimulated by EA 20 min once a day, 6 days a week for 4 weeks. We found that EA treatment downregulated the expression of miR-1b in the local injured nerve. *In vitro* experiments showed that overexpression of miR-1b inhibited the expression of brain-derived neurotrophic factor (BDNF) in rat Schwann cell (SC) line, while BDNF knockdown inhibited the proliferation, migration, and promoted apoptosis of SCs. Subsequently, the rat model of sciatic nerve injury was treated by EA treatment and injection of agomir-1b or antagomir-1b. The nerve conduction velocity ratio (NCV), sciatic functional index (SFI), and S100 immunofluorescence staining were examined and showed that compared with the model group, NCV, SFI, proliferation of SC, and expression of BDNF in the injured nerves of rats treated with EA or EA + anti-miR-1b were elevated, while EA + miR-1b was reduced, indicating that EA promoted sciatic nerve function recovery and SC proliferation through downregulating miR-1b. To summarize, EA may promote the proliferation, migration of SC, and nerve repair after PNI by regulating miR-1b, which targets BDNF.

## Introduction

Peripheral nerve injury (PNI) brings great pain and inconvenience for patients. Electroacupuncture (EA) treatment can effectively relieve neuropathic pain (Zhang et al., 2014; Wang et al., 2018), ameliorate functional indicators, and promote regeneration and anastomosis of the injured nerve within a short term (≤4 weeks) (Zhang et al., 2018). Because of its curative effect and low side effects, EA treatment of PNI has been widely used in clinical practice; therefore, the mechanism of EA has attracted much attention.

MicroRNAs (miRNAs) account for 2–3% of the total number of human genes, which mainly mediate gene silencing by binding to the 3′ untranslated regions (3′ UTR) of target miRNAs (Wanke et al., 2018), make up an important and significant fraction of the regulation of various signaling pathways and biological processes in the repair of PNI (Treiber et al., 2019). Recent researches suggested that miRNAs play an important role in PNI; hence, they have attracted increasing interest in peripheral nerve regeneration field (Yu et al., 2015).

Schwann cells (SCs) are the major glial cells in the peripheral nervous system. After PNI, proliferation and migration of SCs lead to axonal regeneration and secretion of various neurotrophic factors (NTFs), such as nerve growth factor (NGF) and brain-derived neurotrophic factor (BDNF), which are useful for maintaining injured neuron survival, promoting nerve fiber regeneration and new synapse formation (Dai et al., 2013).

As one of the most important members of NTFs, sustained release of exogenous BDNF in the injured peripheral nerve could be a useful tool to increase the axonal density (Lopes et al., 2017). Studies have demonstrated that BDNF is actively involved in the promotion of motor neuron regeneration and motor function recovery (Santos et al., 2016); thus, BDNF plays an essential role in neuronal survival, maintenance, and regeneration after PNI. We have proven that miR-1b overexpression suppressed proliferation and migration of RSC96 and increased cell apoptosis (Liu Y.P. et al., 2018). This study investigated the mechanism of EA promoting the repair of PNI through regulating proliferation, migration, and apoptosis of SCs by miR-1b-mediated BDNF, and providing a theoretical basis for the further study of molecular mechanisms of PNI regeneration.

## Materials and Methods

### Animal Model and Tissue Preparation

SPF-grade Male Wistar rats (body weight 200 ± 20 g) were provided by the Animal Experimental Center of Shanghai University of Traditional Chinese Medicine. The rats were randomly divided into the control group, model group, and EA group (each group contains 10 rats). After anesthesia with sodium pentobarbital (45 mg/kg), the right lower extremity skin of rats in the model group and EA group was disinfected, and posterior lateral thigh incision was performed with a scalpel. The sciatic nerve was surgically exposed under a stereomicroscope, and the sciatic nerve was cut off about 5 mm with a double-sided blade. Two stumps of sciatic nerves were inserted into 12-mm silicone tubes with 2 mm of each stump to form an 8-mm regeneration chamber. The bilateral nerve epithelium was sutured with a 9-0 silk thread to fix it in the silicone tube, then the wound was washed with sterile saline, and the skin was sutured by 3-0 silk threads (Supplementary Figure S3). This study was approved by the animal ethics committee of Shanghai University of TCM and the Animal Research Committee of Shanghai. All of the protocols were based on the “Guide for the Care and Use of Laboratory Animals” of the National Institutes of Health (United States).

### EA Treatment

One day after modeling, the EA group rats were treated with EA, the acupoints “Huantiao” (GB30, the posterior superior edge of the hind limb hip joint) and “Zusanli” (ST36, 1.5 cm below the knee joint) were stimulated for 20 min per day by 0.25 × 13-mm needles, which were connected to G6805A electroacupuncture apparatus through electrodes. The positive pole was connected to GB30 and the negative pole to ST36; then an intermittent wave was set up, and the slight hindlimb muscle contraction was regarded as an appropriate-stimulation degree. The EA treatment process was continued for 4 weeks with a rest every 7 days.

### Quantitative Real-Time RT-PCR (qRT-PCR)

Total RNA of the injured sciatic nerve was extracted using Trizol (Life technologies, Carlsbad, CA, United States). The RNA samples were reversely transcribed using miRcute Plus miRNA First-Strand cDNA Synthesis kit (Tiangen, Beijing, China). The primers of miR-1b and U6 were designed and synthetized by Tiangen Biochemical Technology. The expression of miR-1b normalized to U6 was determined using the miRcute Plus miRNA qPCR Detection Kit (Tiangen) following the amplification protocol: 95°C, 15 min (1 cycle); 94°C, 20 s, 60°C, 34 s (40 cycles).

### PCR

To verify the target gene of miR-1b, cDNA was prepared from total RNA using the FastQuant RT Kit (Tiangen). The sequences of BDNF primers were as follows: GGTTCGAGAGGTCTG ACGAC, GCTGTGACCCACTCGCTAAT. Glyceraldehyde-3- phosphate dehydrogenase (GAPDH), the housekeeping gene, was used to adjust the gene expression of BDNF. The amplification protocol was: 95°C, 5 min (1 cycle); 95°C, 30 s, 52°C, 30 s, 72°C, 1 min (45 cycles); 72°C, 10 min (one cycle). The products were separated by electrophoresis on a 0.8% ethidium bromide-stained agarose gel and visualized by ultraviolet illumination.

### Cell Culture and Transfection

RSC96 cells were purchased from the Institute of Cell Research, Shanghai Academy of Sciences. The cells were cultured in a cell culture incubator with 37°C and 5% CO_2_. RSC96 were transfected with miR-1b mimic or BDNF siRNA (GenePharma, Shanghai, China), respectively, using Lipofectamine 3000 (Thermo, MA, United States) according to the manufacturer’s instructions. The sequences of siRNA duplexes for BDNF were as follows: 5′-CCAGGAGCGUGACAACAAUTT-3′ (sense), 5′-AUUGUUGUCACGCUCCUGGTT-3′ (antisense).

### Western Blot

Forty-eight hours after cell transfection, the total protein was extracted, and the concentration was measured by a BCA protein assay kit (Pierce, Rockford, IL, United States). After denaturation at 95°C for 5 min, equal amounts of protein (40 μg) were separated on 10% SDS-polyacrylamide gel electrophoresis and then transferred into the polyvinylidene fluoride (PVDF) membranes (Millipore, United States). Membranes were incubated with BDNF antibody (Abcam, Cambridge, MA, United States) overnight at 4°C, and then probed with horseradish peroxidase-conjugated secondary antibody (Cell Signaling Technology, Beverly, United States). After being visualized using an enhanced chemiluminescence reagent (Millipore, Billerica, United States), the relative intensities of protein bands were quantified and normalized to GAPDH using ImageJ software (NIH, United States).

### Immunofluorescence Staining

Frozen sections of sciatic nerve tissue or RSC96, which had been fixed at 48 h after transfection were blocked with 10% goat serum and incubated with anti-BDNF (1:100; Abcam) or S100 (1:200; Proteintech, Chicago, United States) at 4°C overnight. After washing with PBS, the samples were incubated with Alexa Fluor 488-conjugated goat anti-rabbit IgG (Cell Signaling Technology, Beverly, United States) for 1 h at room temperature and counterstained with Hoechst 33342 (Biyuntian Biotechnology, Shanghai, China) or 4′,6-diamidino-2-phenylindole (DAPI). The immunostained samples were observed and photographed under a fluorescence microscope (IX53, Olympus, Japan).

### CCK-8 Assay

RSC96, which were cultured in 96-well plates, were transfected with miR-1b MC/mimic or BDNF NC/siRNA. The proliferation of RSC96 was detected through CCK-8 kit (Dojido, Kumamoto, Japan) according to the manufacturer’s instruction at 48 h after transfection. Ten microliters of CCK-8 working solution (Dojido) was added to each well, and the plates were placed in the cell incubator for 2 h. The OD value at 450 nm of each well was read on a microplate reader (Synergy 2, BioTek, United States).

### Transwell Migration Assay

At 48 h after being transfected with miR-1b MC/mimic or BDNF NC/siRNA, RSC96 was suspended in DMEM with a density of 10^6^ cells/ml. One hundred microliters of RSC96 containing DMEM was transferred to the top chamber of Transwells with 8-μm pores (Corning, NY, United States). The lower chamber was loaded with 500 μl of complete medium supplied with 10% fetal bovine serum. After incubation at 37°C in 5% CO_2_ for 12 h, the upper cells remaining in the upper membrane were wiped off smoothly with a cotton swab, while the lower cells were stained with 0.1% crystal violet. Then 10 random fields in each group were imaged and counted under a microscope.

### Terminal dUTP Nick-End Labeling Assay (TUNEL)

Apoptosis of RSC96 was evaluated at 48 h after transfection with miR-1b MC/mimic or BDNF NC/siRNA by using the TUNEL assay (Roche, United States) according to the manufacturer’s instructions, and the nuclei were counterstained with DAPI. The degree of apoptosis was determined by TUNEL-positive cells/total number of cells per high microscopic field (×400).

### Drug Delivery

To test the effect of miR-1b impresses EA on the repair of the injured sciatic nerve, the model of the sciatic nerve injury was constructed as mentioned above. The rats were divided into the model group, EA group, EA + miR-1b group, and EA + anti-miR-1b group (each group contains 10 rats). miR-1b agomir or miR-1b antagomir (20 μM) in a solution of Matrigel was slowly injected into the cavity of the silicone tube by using a precooled micropipette during the model construction, and then the surgical incision was closed in a routine way. One hundred microliters of miR-1b agomir or miR-1b antagomir was given through intramuscular injection every 1 week postoperative. At 4 weeks after surgery, all rats were subjected to functional examination (Supplementary Figure S3). EA treatment was the same as mentioned before.

### Recovery of Sciatic Nerve Functions

The electrophysiological study was performed using the RM6240 Biological Signal Collecting System (Chengdu Instrument Factory, Chengdu, China) to record compound motor action potentials (CMAPs). Briefly, the rat was anesthetized with pentobarbital sodium, and the stimulating electrodes were inserted in the proximal and distal of the sciatic nerve, respectively. The recording electrode was placed in the gastrocnemius muscle. Electric stimulation was applied with the intensity of 5–10 V and wave width of 0.2 ms. The nerve conduction velocity (NCV) was calculated by dividing the distance between two stimulating electrodes by the difference in delay between CMAPs evoked by two stimulating electrodes. The recovery rate of NCV (experimental data/control data × 100%) was calculated using the contralateral side as an internal control. The footprint of each rat in each group was collected by an A4 paper and carbon ink. In the measurement, E represents the injured side, and N represents the healthy side; the footprint length PL (distance from the heel to the toe), the toe width TS (the first to the fifth toe line distance), and the intermediate toe distance IT (the second to the fifth toe line distance), and the sciatic nerve function recovery rate (SFR) formula is as follows: SFR = {1 + [−38.3 × (EPL − NPL)/ NPL + 109.5 × (ETS − NTS)/NTS + 13.3 × (EIT − NIT)/NIT − 8.8]/100} × 100%.

### ELISA

The protein of the injured side nerve, the ipsilateral spinal cord, and the serum of each group were collected and measured by a BCA protein assay kit (Pierce, Rockford, IL, United States). The content of BDNF was detected through an ELISA kit (R&D Systems, Minnesota, United States) according to the manufacturer’s instructions. The OD value of each well was read in a microplate reader (Synergy 2, BioTek, United States) at 450 nm, then a standard curve was drawn to obtain a linear regression equation, and the concentration of BDNF was calculated according to the OD value of the sample.

### Statistical Analysis

All data were reported as the means ± standard deviation. The Student’s *t*-test and one-way analysis of variance (ANOVA) with Scheffe’s *post hoc* multiple-comparison analysis were performed for statistical analyses using SPSS 15.0 for Windows (SPSS, Chicago, IL, United States). *P* < 0.05 was considered to be statistically significant.

## Results

### EA Downregulated the Expression of miR-1b in Injured Sciatic Nerve

**FIGURE 1:**
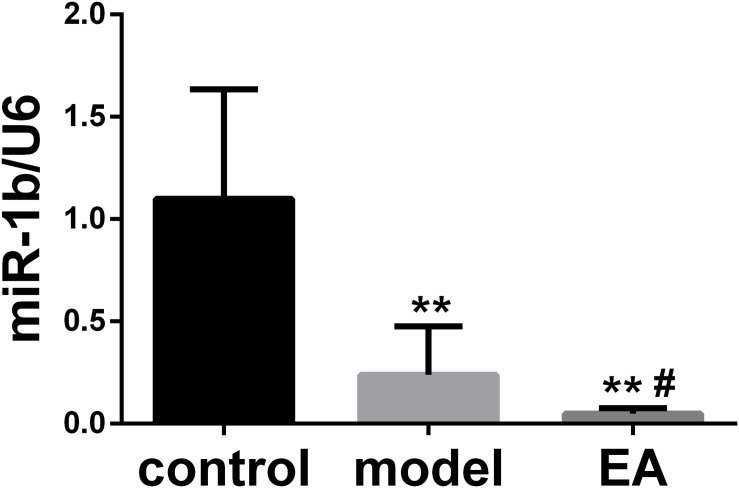
Electroacupuncture (EA) downregulated the expression of miR-1b in the injured sciatic nerve. The expression of miR-1b was detected by quantitative real-time RT-PCR (qRT-PCR). **P < 0.01 versus the control group; ^#^*P* < 0.05 versus the model group.

The expression of miR-1b in the injured sciatic nerve was detected by qRT-PCR. The expression of miR-1b was decreased in both the model group and EA group when compared with the normal control group. The expression of miR-1b in the EA group was much lower than that in the model group (Figure 1). It indicated that EA treatment downregulated the expression of miR-1b in the injured sciatic nerve. Interestingly, our previous results showed that overexpression of miR-1b inhibited the proliferation and migration of SCs and promoted cell apoptosis (Supplementary Figure S1).

### miR-1b Was a Negative Regulator of BDNF

**FIGURE 2:**
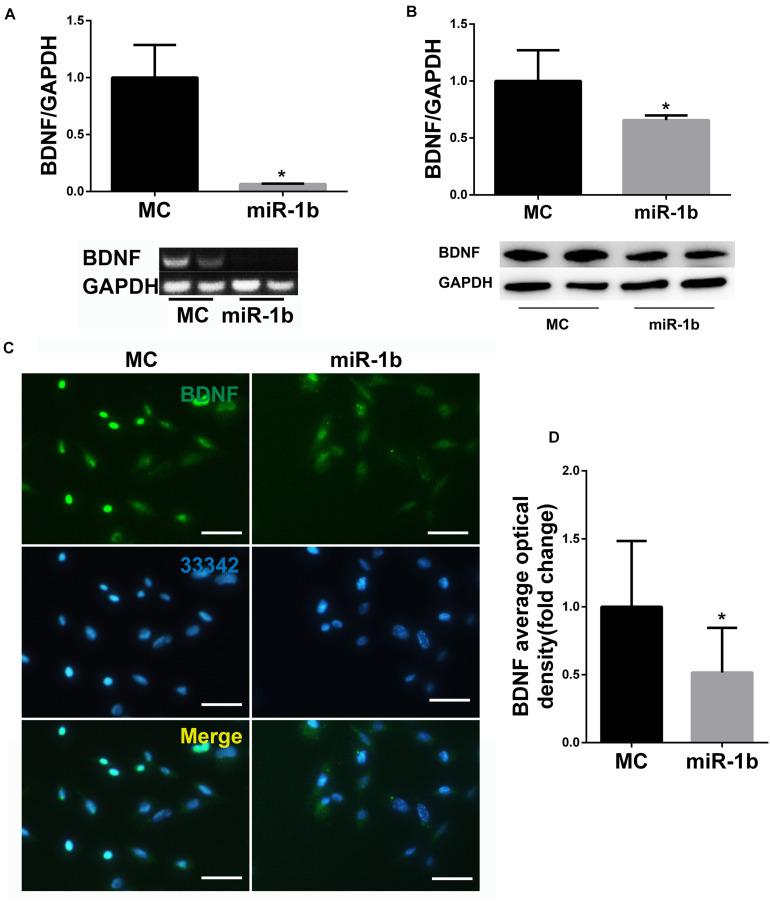
miR-1b targeted brain-derived neurotrophic factor (BDNF) in Schwann cells (SCs). **(A)** BDNF microRNA (miRNA) expression in RSC96 was detected by PCR. *P < 0.05 versus the MC group. **(B)** BDNF protein expression was detected by Western Blot. *P < 0.05 versus the MC group. **(C)** Immunofluorescence staining of BDNF. Scale bar = 25 μm. **(D)** The mean fluorescence optical density of BDNF was analyzed. *P < 0.05 versus the MC group.

After RSC96 was transfected with miR-1b MC or mimic, the mRNA and protein expression levels of BDNF were detected by PCR, Western blot, and immunofluorescence. The expression of BDNF miRNA in the miR-1b overexpression group was dramatically lower compared with the MC group (Figure 2A). The protein level of BDNF in the miR-1b overexpression group was also significantly lower than that in the MC group (Figures 2B–D). These data indicated that miR-1b can regulate the expression of BDNF in SCs.

### Downregulation of BDNF Decreased Proliferation, Migration, and Promoted Apoptosis of SCs

**FIGURE 3:**
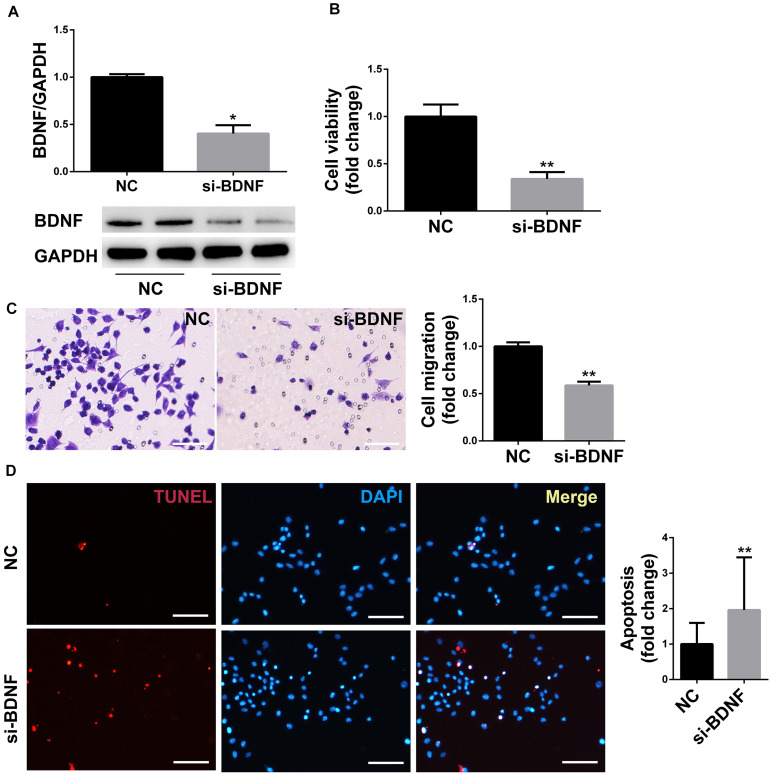
BDNF silencing inhibited the proliferation and migration of SCs and promoted cell apoptosis. **(A)** Western blot was used to detect the protein expression of BDNF in RSC96. *P < 0.05 versus the NC group. **(B)** The cell viability was examined by CCK-8 assay. **P < 0.01 versus the NC group. **(C)** The cell migration was detected by Transwell migration assay. Scale bar = 50 μm. **P < 0.01 versus the NC group. **(D)** TUNEL staining was used to detect cell apoptosis. Scale bar = 50 μm. **P < 0.01 versus the NC group.

The protein expression of BDNF in the siRNA group was decreased compared with the NC group, which confirmed that the gene silencing effect of siRNA was definite (Figure 3A). The cell viability of RSC96 cells was detected by CCK-8 assay, and the migration of RSC96 cells was evaluated by Transwell migration assay. The vitality and migration of SCs in the siRNA group were much lower compared with the NC group (Figures 3B,C), suggesting that the downregulation of the target gene BDNF of miR-1b inhibited SCs proliferation and migration. The apoptosis of RSC96 cells was detected by TUNEL staining, and the cell apoptosis was significantly increased after the downregulation of BDNF (Figure 3D), which indicated that downregulation of BDNF promoted the apoptosis of RSC96.

### EA Promoted Sciatic Nerve Function Recovery and SC Proliferation Through Downregulating miR-1b

**FIGURE 4:**
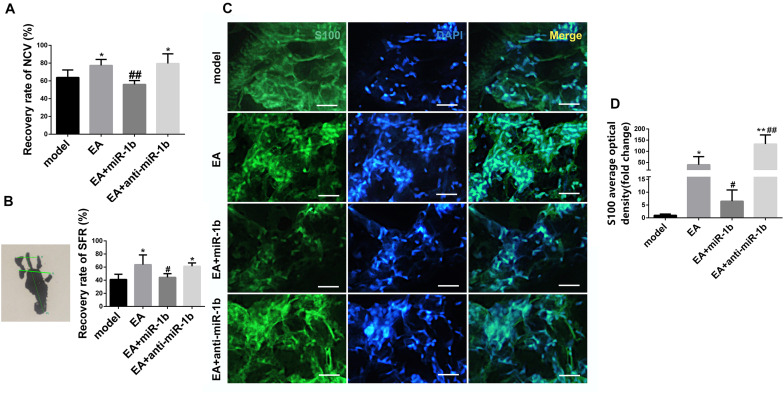
EA promoted sciatic nerve function recovery and SC proliferation through downregulating miR-1b. **(A)** The recovery rate of NCV was compared among the groups. *P < 0.05 versus the model group. ^##^*P* < 0.01 versus the EA group. **(B)** The recovery rate of SFR was compared among the groups. *P < 0.05 versus the model group. ^#^*P* < 0.05 versus the EA group. **(C)** Immunofluorescence staining of S100 in the injured sciatic nerve. Scale bar = 50 μm. **(D)** The mean fluorescence density of S100 was analyzed. *P < 0.05 and **P < 0.01 versus the model group. ^#^*P* < 0.05 and ^##^*P* < 0.01 versus the EA group.

NCV and SFR, which are two sciatic nerve function recovery indexes, were detected at 4 weeks after surgery. The recovery rate of NCV and SFR in the EA group and EA + anti-miR-1b group were higher compared with the model group (Figures 4A,B). However, compared with the EA group, the recovery rate of NCV and SFR in the EA + miR-1b group were significantly decreased, which suggested that EA treatment promoted the recovery of sciatic nerve function after injury, while overexpression of miR-1b attenuated the therapeutic effects exerted by EA treatment. We further observed that the regulation of miR-1b *in vivo* during EA treatment could affect the proliferation of SCs in the injured sciatic nerve. S100 immunofluorescence staining showed that there were more SCs in the EA and EA + anti-miR-1b group compared with the model group. Interestingly, the proliferation of SCs, which was mediated by EA treatment, was abolished after the administration of miR-1b (Figures 4C,D). Besides, EA also promoted myelin and axon regeneration through dowregulating miR-1b (Supplementary Figure S2).

### EA Increased the Expression of BDNF by Downregulating miR-1b

**FIGURE 5:**
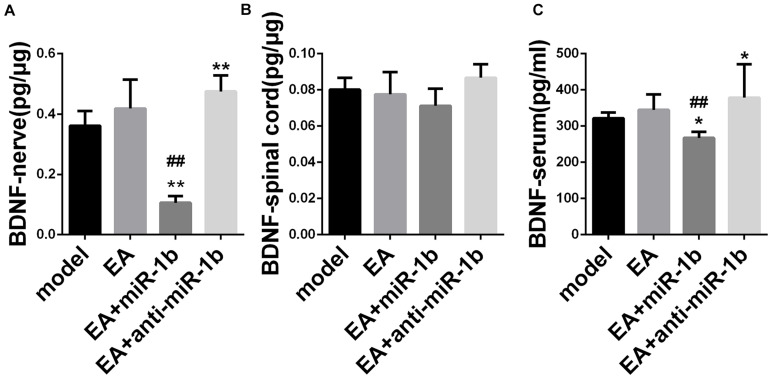
EA increased the expression of BDNF by downregulating miR-1b in injured sciatic nerve and serum. The expression of BDNF expression in the injured sciatic nerve **(A)**, ipsilateral spinal cord **(B)**, and in serum **(C)** was detected by ELISA. *P < 0.05 and **P < 0.01 versus the model group. ^##^*P* < 0.01 versus the EA group.

The expression of BDNF in the injured sciatic nerve, ipsilateral spinal cord, and serum was detected by ELISA. In the injured sciatic nerve and serum, EA had a tendency to increase the level of BDNF. The expression of BDNF was lower in the EA + miR-1b group and higher in the EA + anti-miR-1b group compared with the model group or the EA group. However, changes in BDNF expression in the spinal cord were not noticeable (Figure 5). Overall, these data provided evidence that EA increased the expression of BDNF in injured sciatic nerve and serum through downregulating miR-1b.

## Discussion

miRNAs are a class of short (18–25 nucleotides) non-coding small RNAs, which are widely found in eukaryotes. The discovery of miRNAs ranks first among the world’s top 10 technological breakthroughs in Science in 2002. miRNAs are involved in the regulation of various biological processes including autophagy (Hargarten and Williamson, 2018), tumors (Krump and You, 2018), allergic diseases (Tost, 2018), and T-cell immunity (Giri et al., 2019) for mediating gene silencing. Numerous investigations have shown that miRNAs play a vital role in PNI and peripheral nerve repair; for instance, the expression of miRNAs in dorsal root ganglia (DRG) tissues after sciatic nerve injury in rats was significantly changed (Li et al., 2012). miR-142-3p can target CDKN1B and TIMP3 to enhance the viability of rat DRG neurons and inhibit apoptosis after sciatic nerve injury (Wu D.M. et al., 2018). miR-7 inhibits neural stem cell migration and proliferation by targeting cdc42 and affects repair after PNI (Zhou et al., 2018). miR-221-3p may inhibit SCs to develop into the myelin sheath, which cocultured with the DRG neurons *in vitro* by targeting GF1-A-binding protein 1 (Nab1) (Zhao L. et al., 2018).

miR-1b belongs to the miR-1 family, whose members include miR-1, miR-1b, miR-206, and miR-3571. Researchers have identified miR-1-targeting BDNF in the regulation of SC proliferation and migration after nerve injury (Yi et al., 2016); in addition, miR-1 regulated chronic neuropathic pain in rats through targeting cx43 and BDNF (Neumann et al., 2015), and miR-206 can target BDNF to the regulation of the MERK-ERK signaling pathway to affect neuro stress pain (Sun et al., 2017). The same family of miRNAs is used to be similar in their targets and expression profiles, so the role of miR-1b in PNI and nerve repair deserves further study.

BDNF protein was markedly increased after PNI in the spinal cord and DRG (Sanna et al., 2017), which has emerged as an important modulator of axon regeneration (McGregor and English, 2018). Electrical muscle stimulation elevates intramuscular BDNF following PNI and repair in rats (Willand et al., 2016).

Due to differences in structure and function, damaged axons in the central nervous system (CNS) of adult mammals are usually unable to regenerate, while nerve recovery is generally very poor despite the ability of peripheral nerves to spontaneously regenerate after PNI (Gu et al., 2014). Both electrical stimulation (Balseanu et al., 2020; Wu et al., 2020) and acupuncture (Wong et al., 2011; He et al., 2015) have beneficial effects on PNI (He et al., 2015; Wu et al., 2020) or central nerve injury (CNI) (Wong et al., 2011; Balseanu et al., 2020). EA, which is the combination of electrical stimulation and acupuncture, has therapeutic effects on both PNI (Wu J. et al., 2018) and CNI (Cai et al., 2020). Although EA treatment has been convinced to be helpful for nerve repair after PNI (Inoue et al., 2003; Pan et al., 2019), the mechanism is not very clear. Studies showed that the expression of multiple miRNAs has changed such as miR-129 (Zhu et al., 2018) and miR-195 (Shi et al., 2013) after PNI. It has been reported that EA enhanced neurobehavioral functional recovery against ischemic stroke through targeting of SOX2-mediated axonal regeneration by miR-132 (Cui et al., 2017; Zhao X. et al., 2018). Another research suggested that several miRNAs were involved in chronic electroacupuncture tolerance in the rat hypothalamus (Cui et al., 2017; Zhao X. et al., 2018). Our previous studies have confirmed that EA could promote SC migration and NGF expression in injured local nerve as well as BDNF (Hu et al., 2018). In this study, it was first discovered that miR-1b expression was significantly downregulated in the local nerve after EA. Overexpression of miR-1b in cultured SCs downregulated the expression of BDNF, and overexpression of miR-1b or BDNF gene silencing inhibited the proliferation and migration of SCs and promoted cell apoptosis. *In vivo* experiments revealed that overexpression of miR-1b inhibited the expression of BDNF in the local injured nerve, decreased the proliferation of SCs, and inhibited the effect of EA on the recovery of sciatic nerve function. These data suggested that EA may affect the proliferation, migration, and apoptosis of SCs and promote nerve repair after PNI by regulating miR-1b, which targets BDNF.

However, there are still some limitations to this study. First, *in vitro* cell experiments were performed on SC strains instead of primary SCs. Second, there was a lack of direct observation of morphological parameters in rats. Third, as is well known, PNI is mostly caused by trauma. Body weights of PNI model rats ranged from 180 to 220 g (Li et al., 2015; Zhao et al., 2017), 200 to 250 g (Chang et al., 2016), 250 to 300 g (Zarbakhsh et al., 2016), etc. The rats’ body weight in this study is 260 ± 20 g after being adaptive fed for 1 week before modeling, which was considered to be a suitable weight. Stroke is the main type of CNS damage and is closely related to age (Petcu et al., 2008), and the age factor is also related to the repair of PNI (Kovačič et al., 2009; Liu J. et al., 2018), thus using aged animals is much more relevant, which is the limitation of our research.

In addition, it is worth optimizing the delivery method of miRNA inhibitor or mimic *in vivo*.

## Conclusion

Our experimental results showed that miR-1b expression was downregulated in the local nerve after EA, and miR-1b can target BDNF. Overexpression of miR-1b or knock-down of BDNF can inhibit the proliferation and migration of SCs and promote their apoptosis. Furthermore, EA promoted sciatic nerve function recovery through downregulating miR-1b after PNI. This study provided a basis and a new direction for the study of PNI repair mechanisms and the clinical application of EA in the treatment of PNI.

## Ethics Statement

The animal study was reviewed and approved by this study was approved by the animal ethics committee of Shanghai University of TCM and the Animal Research Committee of Shanghai. All of the protocols were based on the “Guide for the Care and Use of Laboratory Animals” of the National Institutes of Health (USA).

## Author Contributions

HG and SS contributed to the experimental design, data interpretation, and editing of the manuscript. Y-PL performed the surgery, plasmid construction and luciferase assay, cell culture, animal experiments, and wrote the manuscript. ZL and CW performed the RT-PCR, WB, and ELISA experiment. HC, HL, and TZ performed the surgery, IF, and data analysis. Y-PL, ZL, and CW contributed to the EA experiment, data analysis, and interpretation. All authors contributed to the article and approved the submitted version.

## Conflict of Interest

The authors declare that the research was conducted in the absence of any commercial or financial relationships that could be construed as a potential conflict of interest.

## Supplementary Material

Supplementary Figure 1 Overexpression of miR-1b inhibited the proliferation and migration of SCs and promoted cell apoptosis. **(A)** The cell viability was examined by CCK-8 assay. ^∗∗^*P* < 0.01 versus the MC group. **(B)** The cell migration was detected by Transwell migration assay. Scale bar = 25 μm. ^∗∗^*P* < 0.01 versus the MC group. **(C)** TUNEL staining was used to detect cell apoptosis. Scale bar = 50 μm. ^∗∗^*P* < 0.01 versus the MC group.

Supplementary Figure 2 EA promoted myelin and axon regeneration through down-regulating miR-1b. **(A)** General observation of nerve cross-sectional structure by Masson trichrome staining. The nerve membrane which was rich in collagen fibers was stained blue, and the cytoplasm was stained red. Scale bar = 25 μm. **(B)** Detection of myelin regeneration by immunohistochemical staining of myelin basic protein (MBP). Scale bar = 25 μm. **(C)** Detection of axon regeneration by immunofluorescence staining of Neurofilament Heavy (NF200). Scale bar = 25 μm.

Supplementary Figure 3 Rat drug administration timeline, modeling photos and general observation. **(A)** Timeline for the experimental design. **(B)** Modeling photos: (1) The sciatic nerve was dissociated. (2) The sciatic nerve was cut off. (3) The silicone tube was connected to the nerve ends. (4) The outer nerve membrane was sutured with silk thread. (5) The skin was sutured. (6) EA treatment. **(C)** Observation of the general condition of the affected foot of rat.
