# Supplementary figures and images for: Electroacupuncture Promoted Nerve Repair After Peripheral Nerve Injury by Regulating miR-1b and Its Target Brain-Derived Neurotrophic Factor
Source: Front Neurosci. 2020 Sep 29;14:525144. doi: 10.3389/fnins.2020.525144 (PMC7550428; doi:10.3389/fnins.2020.525144)

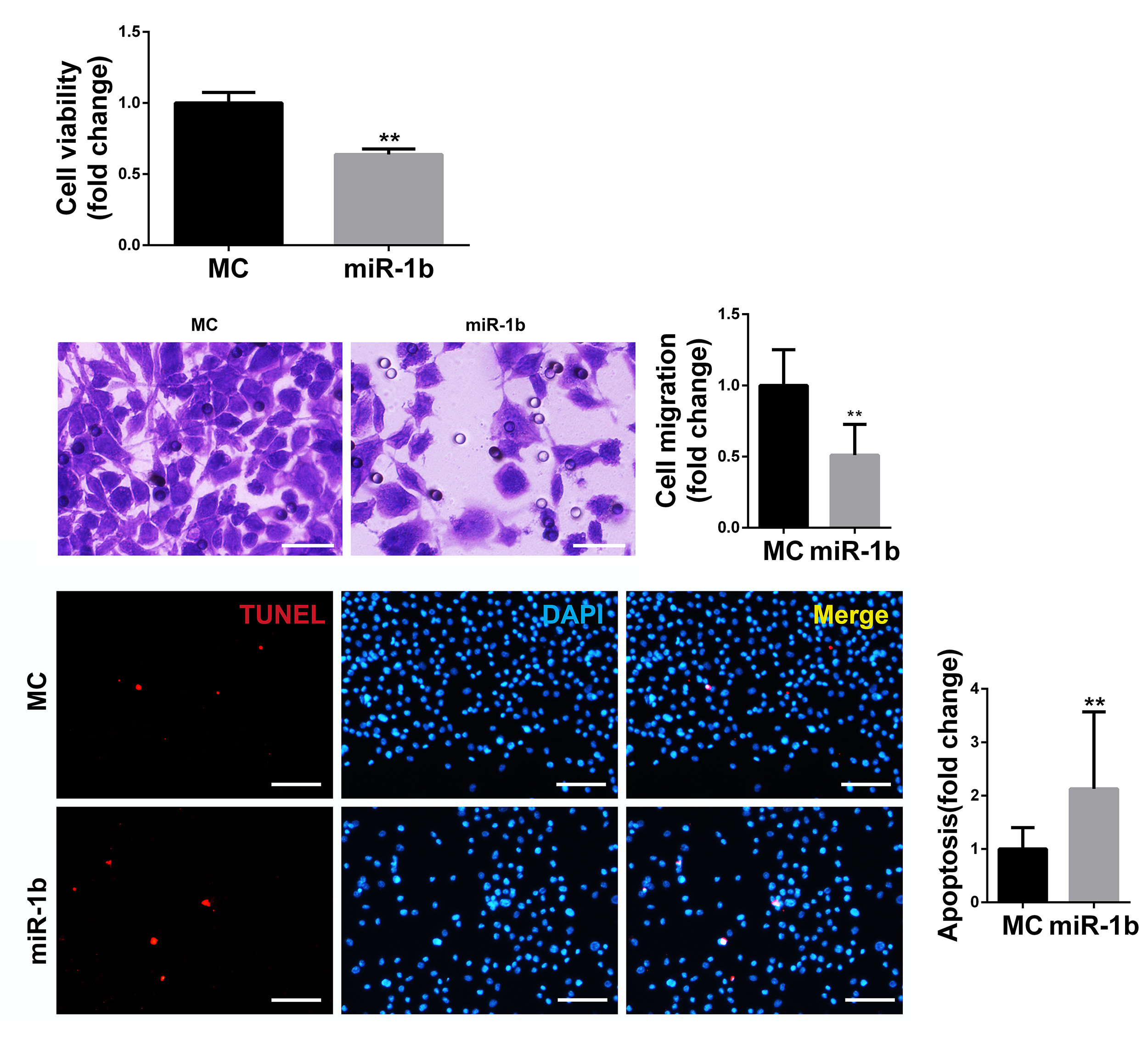

Supplement: Supplementary Figure 1 — Overexpression of miR-1b inhibited the proliferation and migration of SCs and promoted cell apoptosis. (A) The cell viability was examined by CCK-8 assay. ∗∗P < 0.01 versus the MC group. (B) The cell migration was detected by Transwell migration assay. Scale bar = 25 μm. ∗∗P < 0.01 versus the MC group. (C) TUNEL staining was used to detect cell apoptosis. Scale bar = 50 μm. ∗∗P < 0.01 versus the MC group. [file Image_1.TIF]

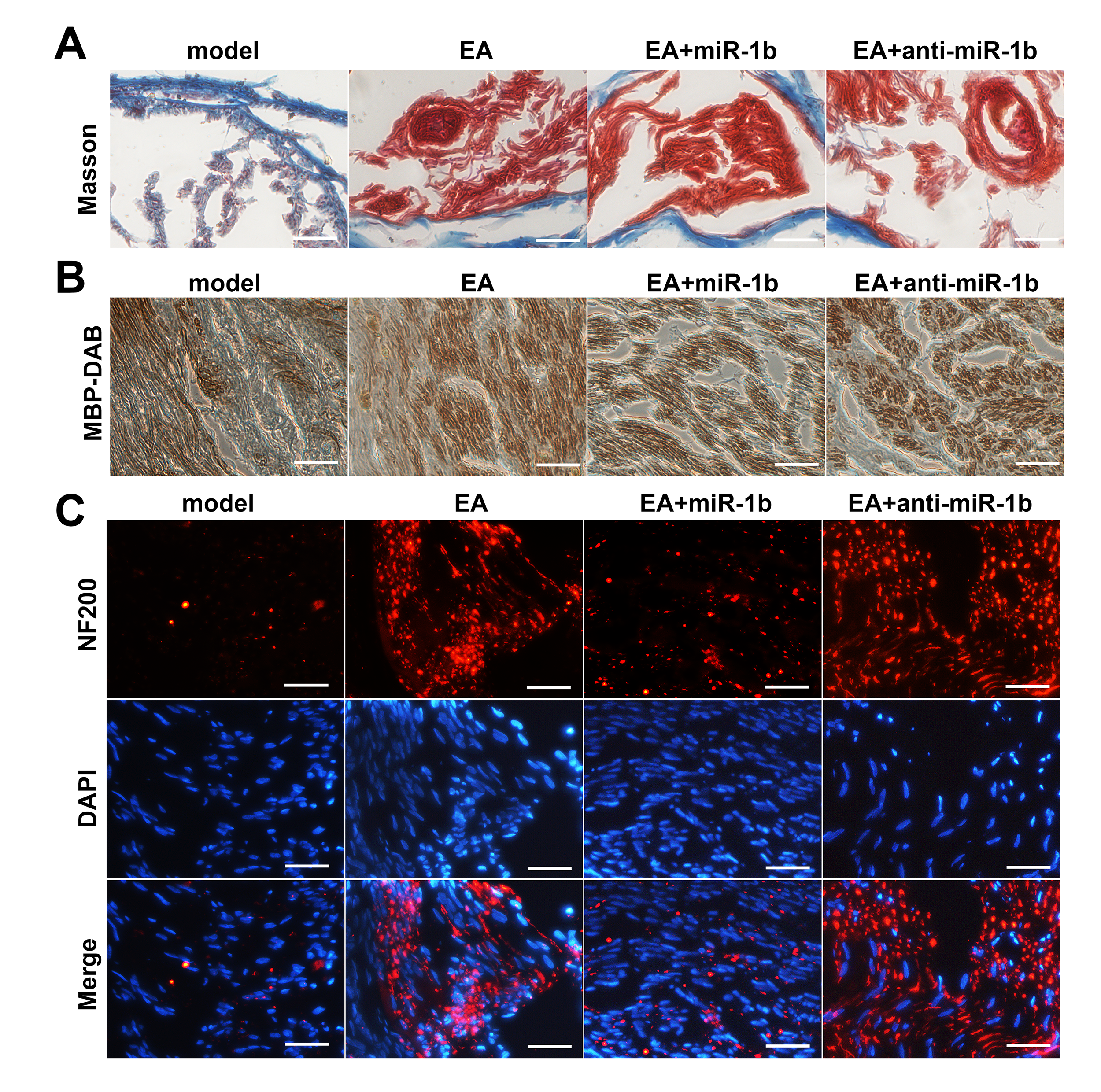

Supplement: Supplementary Figure 2 — EA promoted myelin and axon regeneration through down-regulating miR-1b. (A) General observation of nerve cross-sectional structure by Masson trichrome staining. The nerve membrane which was rich in collagen fibers was stained blue, and the cytoplasm was stained red. Scale bar = 25 μm. (B) Detection of myelin regeneration by immunohistochemical staining of myelin basic protein (MBP). Scale bar = 25 μm. (C) Detection of axon regeneration by immunofluorescence staining of Neurofilament Heavy (NF200). Scale bar = 25 μm. [file Image_2.TIF]

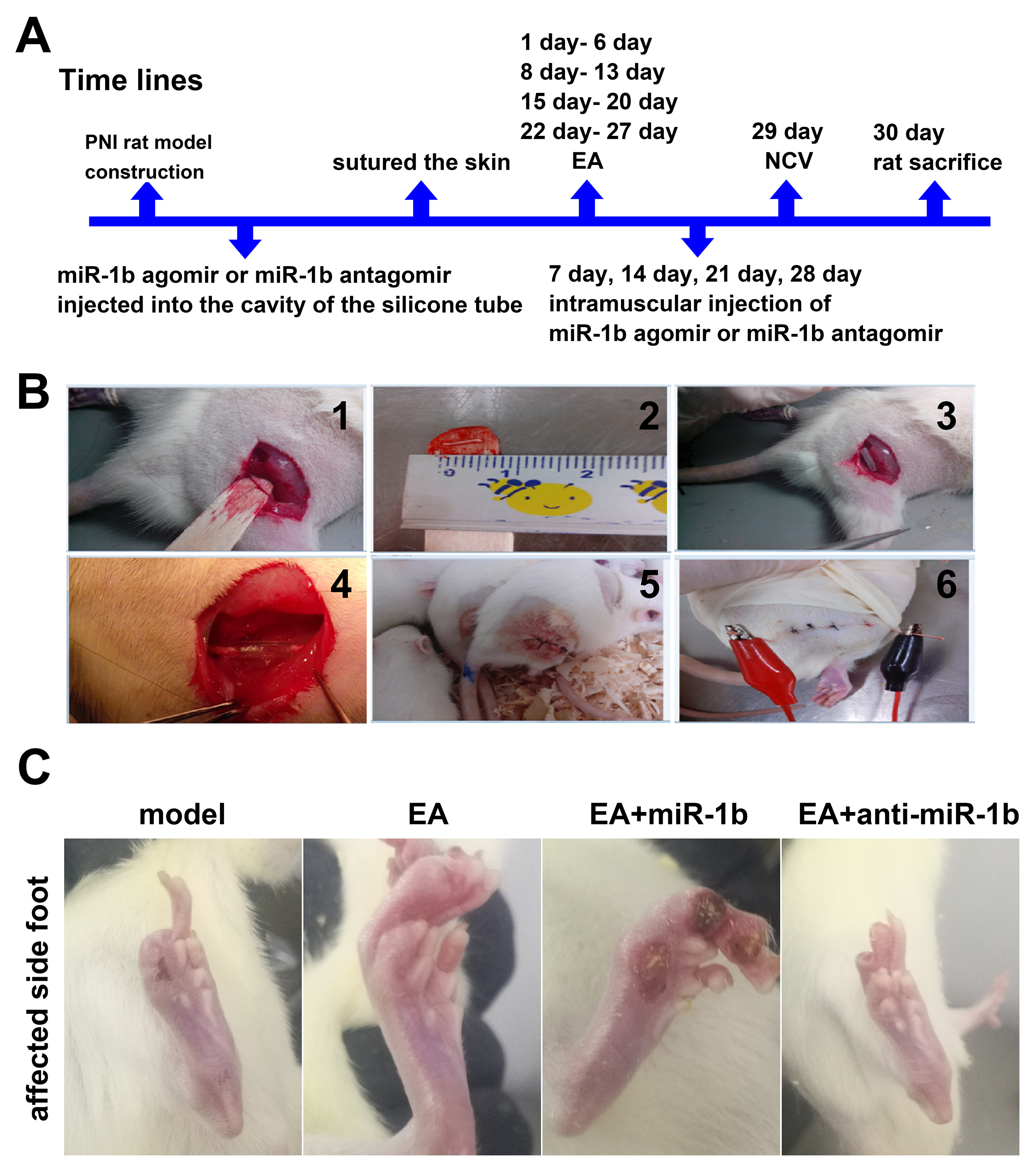

Supplement: Supplementary Figure 3 — Rat drug administration timeline, modeling photos and general observation. (A) Timeline for the experimental design. (B) Modeling photos: (1) The sciatic nerve was dissociated. (2) The sciatic nerve was cut off. (3) The silicone tube was connected to the nerve ends. (4) The outer nerve membrane was sutured with silk thread. (5) The skin was sutured. (6) EA treatment. (C) Observation of the general condition of the affected foot of rat. [file Image_3.TIF]
